# Nourin-Associated miRNAs: Novel Inflammatory Monitoring Markers for Cyclocreatine Phosphate Therapy in Heart Failure

**DOI:** 10.3390/ijms22073575

**Published:** 2021-03-30

**Authors:** Salwa A. Elgebaly, Robert Todd, Donald L. Kreutzer, Robert Christenson, Nashwa El-Khazragy, Reem K. Arafa, Mostafa A. Rabie, Ahmed F. Mohamed, Lamiaa A. Ahmed, Nesrine S. El Sayed

**Affiliations:** 1 Research & Development, Nour Heart, Inc., Vienna, VA 22180, USA; 2 Department of Surgery, School of Medicine, UConn Health, Farmington, CT 06032, USA; 3 Chemistry Department, ProChem Intl, LLC, Sheboygan, WI 53085, USA; robert.todd@hotmail.com; 4 Department of Surgery, School of Medicine, and Cell & Molecular Tissue Engineering, UConn Health, LLC, Farmington, CT 06032, USA; kreutzer@uchc.edu; 5 Department of Pathology, School of Medicine, University of Maryland, Baltimore, MD 21201, USA; RCHRISTENSON@umm.edu; 6 Department of Clinical Pathology-Hematology, Ain Shams Medical Research Institute (MASRI), Faculty of Medicine, Ain Shams University, Cairo 11566, Egypt; nashwaelkhazragy@med.asu.edu.eg; 7 Biomedical Sciences Program & Drug Design and Discovery Lab, Zewail City of Science and Technology, Cairo 12578, Egypt; rkhidr@zewailcity.edu.eg; 8 Department of Pharmacology and Toxicology, Faculty of Pharmacy, Cairo University, Cairo 11562, Egypt; mostafa.mohammed@pharma.cu.edu.eg (M.A.R.); ahmed.fathi@pharma.cu.edu.eg (A.F.M.); lamiaa.ahmed@pharma.cu.edu.eg (L.A.A.); nesrine.salah@pharma.cu.edu.eg (N.S.E.S.)

**Keywords:** heart failure, Nourin, inflammatory mediators, *miR-137* and *miR-106b-5p*, myocardial ischemia, monitoring biomarker, drug therapy response, cyclocreatine phosphate, cardioprotection

## Abstract

Background: Cyclocreatine phosphate (CCrP) is a potent bioenergetic cardioprotective compound known to preserve high levels of cellular adenosine triphosphate during ischemia. Using the standard Isoproterenol (ISO) rat model of heart failure (HF), we recently demonstrated that the administration of CCrP prevented the development of HF by markedly reducing cardiac remodeling (fibrosis and collagen deposition) and maintaining normal ejection fraction and heart weight, as well as physical activity. The novel inflammatory mediator, Nourin is a 3-KDa formyl peptide rapidly released by ischemic myocardium and is associated with post-ischemic cardiac inflammation. We reported that the Nourin-associated *miR-137* (marker of cell damage) and *miR-106b-5p* (marker of inflammation) are significantly upregulated in unstable angina patients and patients with acute myocardial infarction, but not in healthy subjects. Objectives: To test the hypothesis that Nourin-associated *miR-137* and *miR-106b-5p* are upregulated in ISO-induced “HF rats” and that the administration of CCrP prevents myocardial injury (MI) and reduces Nourin gene expression in “non-HF rats”. Methods: 25 male Wistar rats (180–220 g) were used: ISO/saline (*n* = 6), ISO/CCrP (0.8 g/kg/day) (*n* = 5), control/saline (*n* = 5), and control/CCrP (0.8 g/kg/day) (*n* = 4). In a limited study, CCrP at a lower dose of 0.4 g/kg/day (*n* = 3) and a higher dose of 1.2 g/kg/day (*n* = 2) were also tested. The Rats were injected SC with ISO for two consecutive days at doses of 85 and 170 mg/kg/day, respectively, then allowed to survive for an additional two weeks. CCrP and saline were injected IP (1 mL) 24 h and 1 h before first ISO administration, then daily for two weeks. Serum CK-MB (U/L) was measured 24 h after the second ISO injection to confirm myocardial injury. After 14 days, gene expression levels of *miR-137* and *miR-106b-5p* were measured in serum samples using quantitative real-time PCR (qPCR). Results: While high levels of CK-MB were detected after 24 h in the ISO/saline rats indicative of MI, the ISO/CCrP rats showed normal CK-MB levels, supporting prevention of MI by CCrP. After 14 days, gene expression profiles showed significant upregulation of *miR-137* and *miR-106b-5p* by 8.6-fold and 8.7-fold increase, respectively, in the ISO/saline rats, “HF rats,” compared to the control/saline group. On the contrary, CCrP treatment at 0.8 g/kg/day markedly reduced gene expression of miR-137 by 75% and of *miR-106b-5p* by 44% in the ISO/CCrP rats, “non-HF rats,” compared to the ISO/Saline rats, “HF rats.” Additionally, healthy rats treated with CCrP for 14 days showed no toxicity in heart, liver, and renal function. Conclusions: Results suggest a role of Nourin-associated *miR-137* and *miR-106b-5p* in the pathogenesis of HF and that CCrP treatment prevented ischemic injury in “non-HF rats” and significantly reduced Nourin gene expression levels in a dose–response manner. The Nourin gene-based mRNAs may, therefore, potentially be used as monitoring markers of drug therapy response in HF, and CCrP—as a novel preventive therapy of HF due to ischemia.

## 1. Introduction

Heart failure is becoming an increasing concern to healthcare worldwide because of the increasing disease burden and economic impact. Its clinical diagnosis is when the heart fails to provide sufficient circulatory force to meet the body’s metabolic requirements. It is caused by insufficient oxygenation due to a decrease in blood supply, a condition which is directly linked to myocardial injury and coronary artery occlusion [1]. As the population continues to age, it is expected that the prevalence of this disease will continue to rise. Due to the increase trend in the incidence of HF, recent studies have focused on the “treatment” and “preventive” therapies of the disease.

Hyperactivity of the immune system contributes to the progress of heart failure. Several pro-inflammatory cytokines, including tumor necrosis factor alpha (TNF-α) [2], interleukin 6 (IL-6), and interleukin 1β (IL-1β) exacerbate the hemodynamic inflammatory process, thus, exert direct cardiotoxicity and contribute to reduced cardiac contractile function. In addition, endotoxin was reported to activate proinflammatory cytokines in HF [3]. Inflammation-induced cardiomyocyte structural deterioration in HF results from leukocyte extravasation which is maintained by the action of adhesion molecules, where integrins and selectins mediate cell-to-cell interaction and promote leukocyte adhesion to vascular endothelial cells (VECs). Especially, lymphocyte function-associated antigen (LFA-1) and glycoprotein IIb/IIIa are found to roll out leukocytes on the vascular endothelium, a function that is mediated by leukocyte (L) selectin and platelet (P) selectin [4]. Previous studies have also demonstrated the upregulation of plasma-soluble adhesion molecules ICAM and VCAM in patients with HF [5].

Elgebaly et al. [6,7,8,9,10,11,12,13,14] reported that Nourin is a potent inflammatory mediator rapidly released by human and animal hearts in response to reversible ischemic injury and necrosis. Nourin is a 3-KDa N-formyl peptide and its release by ischemic cardiac tissue is associated with post-ischemic cardiac inflammation in early ischemia/reperfusion animal models of cardiopulmonary bypass surgery and acute myocardial infarction (AMI) [6]. Nourin was purified from cardioplegic solutions collected during cardiac arrest from patients undergoing cardiopulmonary bypass for coronary revascularization [7], and its amino acid sequence was determined. As a potent inflammatory mediator, Nourin stimulates leukocyte chemotaxis and vascular endothelial cell migration and activates human monocytes, neutrophils, and VECs to express high levels of cytokine storm mediators, digestive enzymes, and free radicals, including TNF-α (a key stimulant of apoptosis), IL-1β, IL-8, LECAM-1, ICAM-1, and ELAM-1, as well as type IV collagenase, N-acetyl-β-glucosaminidase, gelatinases, and superoxide anion [6]. Accordingly, Nourin can be characterized as an alarmin that promotes the innate immune response since it is rapidly released by local myocardial tissues following ischemia and contributes to the initiation and amplification of post-reperfusion myocardial inflammation. Therefore, Nourin can be a diagnostic and therapeutic target to develop laboratory tests to diagnose and monitor myocardial ischemia/necrosis, as well as therapies to control early and late post-reperfusion inflammation and injury.

Demand ischemia causes irreversible myocardial injury through exhaustion of cellular adenosine triphosphate (ATP). CCrP is a potent bioenergetic cardioprotective compound which preserves high levels of ATP during ischemia [6,15]. Elgebaly et al. [6,9,12] demonstrated that the administration of cyclocreatine (CCr) and its water-soluble compound CCrP prevented myocardial ischemic injury, reduced post-ischemic cardiac inflammation and myocardial apoptosis, as well as restored a strong contractile function immediately after reperfusion in animal models of AMI, global warm cardiac arrest, cardiopulmonary bypass, heart transplantation, and heart failure. Specifically, using a standard ISO rat model of heart failure, we recently reported that the administration of CCrP at 0.8 g/kg/day for 14 days prevented ischemic injury and the subsequent development of heart failure by markedly reducing cardiac remodeling by over 80% (fibrosis and collagen deposition) and maintaining normal ejection fraction, heart weight, as well as physical activity [9]. Thus, CCrP is a promising first-in-class cardioprotective drug which may potentially be used for preventive therapy against the development of HF due to ischemia. The US Food and Drug Administration (FDA) has recently awarded CCrP the orphan drug status with the following unique designation: “Prevention of Ischemic Injury to Enhance Cardiac Graft Recovery and Survival in Heart Transplantation” (DRU-2015-4951).

**Figure 1:**
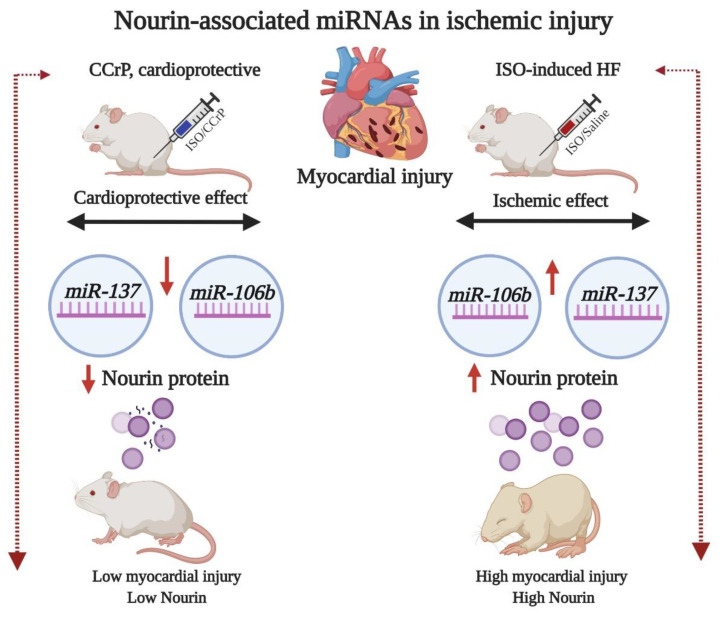
A flow diagram at day 14 illustrating that ischemia-induced ISO rats which developed HF show upregulation of Nourin-associated *miR-137* (marker of cell damage) and *miR-106b-5p* (marker of inflammation) with a likely increase in translation and production of the Nourin protein. The diagram also indicates amelioration of myocardial ischemic injury and the molecular regulation of the Nourin protein when rats were treated with the cardioprotective compound, CCrP, in rats that did not develop HF. ISO: isoproterenol, CCrP: cyclocreatine phosphate.

Since myocardial ischemia is a major denominator of heart failure and we have recently demonstrated that the Nourin-associated gene expression of *miR-137* (marker of cell damage) and *miR-106b-5p* (marker of inflammation) is significantly upregulated in unstable angina (UA) and acute ST elevation myocardial infarction (STEMI) patients [10,11,14], we examined the hypothesis that the gene expression of Nourin miRNAs *miR-137* and *miR-106b-5p* is also upregulated at day 14 in ISO/saline “HF rats” and that the administration of CCrP reduces myocardial injury and the expression levels of Nourin miRNAs in ISO/CCrP “non-HF rats.” Figure 1 indicates that ISO-induced myocardial ischemic injury resulted in upregulation of *miR-137* and *miR-106b-5p* with a likely increase of translation and production of the Nourin protein, as we had previously demonstrated using the intact canine models of AMI and bypass surgery, where the circulating Nourin protein was markedly elevated [6]. Furthermore, CCrP administration resulted in reduction of ISO-induced myocardial injury and significantly inhibited gene expression levels of *miR-137* and *miR-106b-5p* with a likely reduction of the Nourin level, as we had previously demonstrated in the intact canine models of AMI and cardiopulmonary bypass surgery, where the circulating Nourin protein was markedly reduced after CCrP treatment [6,12]. Based on these results, Nourin miRNAs might play a role in the pathogenesis of heart failure due to ischemic injury, while CCrP treatment prevents myocardial damage and reduces their gene expression.

## 2. Results

### 2.1. Cyclocreatine Phosphate Prevents Myocardial Ischemic Injury and Safety Studies

**Figure 2:**
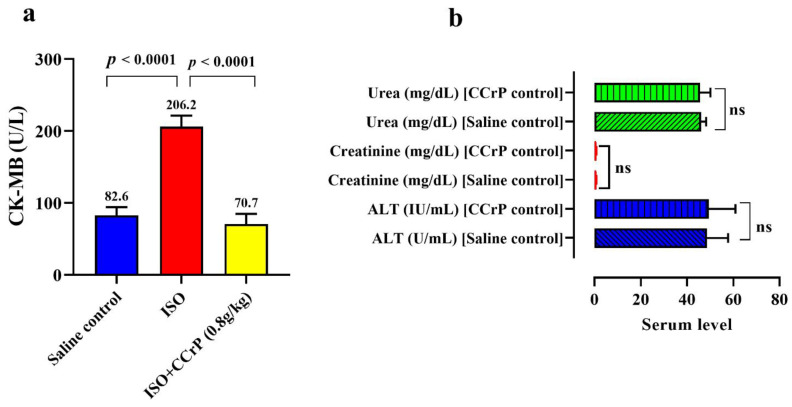
(**a**) Cardioprotective effect of CCrP in the ISO/CCrP “non-HF rats” as indicated by the low levels of the cardiac marker CK-MB (U/L) compared to the high elevated levels of ISO/saline “HF rats.” (**b**) Safety of CCrP at a dose of 0.8 g/kg/day for 14 days was studied by IP injecting CCrP daily to healthy rats. There was no significant difference between normal rats treated with saline or CCrP for the levels of liver enzyme ALT, kidney creatinine, and urea. CCrP showed no toxicity in liver and renal function. ISO: isoproterenol, CCrP: cyclocreatine phosphate, CK-MB: creatine kinase isoenzyme MB, and ALT: alanine transaminases.

**Figure 3:**
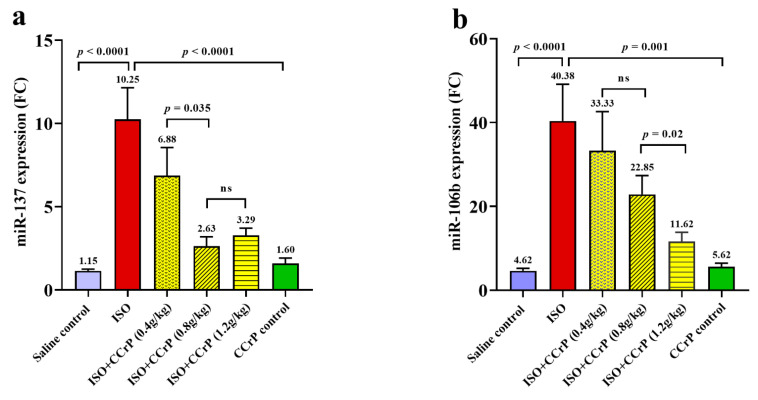
The inhibitory effect of CCrP on gene expression on Nourin-associated *miR-137* (**a**) and *miR-106b-5p* (**b**) in experimental groups as expressed in mean values ± SD. The dose–response manner indicates a significant difference between groups of ISO/CCrP “non-HF rats” and ISO/saline “HF rats” (*p* < 0.05). Non-significant difference is indicated by *p*-value > 0.05. FC: fold count, miRNAs are expressed as fold change compared to the expression in the saline control group (negative control/saline), ISO: isoproterenol, ns: non-significant, CCrP: cyclocreatine phosphate.

Serum creatine kinase-MB (CK-MB) (U/L) was measured 24 h after the last ISO injection to confirm development of myocardial injury (Figure 2a). The saline-treated ISO rats (ISO/saline) had very high levels of CK-MB of 206.20 ± 15.3 U/L, with a 2.49-fold increase over the control/saline rats (82.6 ± 11.6 U/L) (*p* < 0.0001), indicative of the presence of myocardial injury. On the other hand, CCrP-treated ISO (ISO/CCrP) rats showed low levels of CK-MB (71 ± 14.2 U/L) (*p* < 0.0001), which were comparable to the baseline healthy/saline rats. The absence of elevation of CK-MB in the ISO/CCrP rats confirms our previous findings that CCrP treatment prevents ischemic injury and maintains healthy hearts [6,9,12]. Furthermore, we determined if there is any toxic effect of CCrP therapy at 0.8 g/kg/day for 14 days on renal and liver function. The results revealed no significant difference for the serum level of the liver necrosis marker, alanine transaminase (ALT), blood urea, and serum creatinine between the saline control and the CCrP control group (Figure 2b). Additionally, the lack of effect of CCrP on the Nourin miRNA gene expression by cardiac tissue (Figure 3a,b) is supported by the absence of toxicity of CCrP on liver (ALT) and kidney (urea, creatinine) function. Thus, results of this limited safety study suggest that CCrP has no toxicity in heart, liver, and renal function.

### 2.2. Gene Expression Levels of Nourin-Associated miR-137 and miR-106b-5b

Using the Nourin amino acid sequence and in silico integrative bioinformatics analysis as well as gene ontology, the Nourin-associated gene miRNAs were selected. The results revealed that the two miRNAs, *miR-137* and *miR-106b-5b*, represent the molecular regulatory gene for Nourin in myocardial ischemia [10,11,14]. Nourin-associated miRNAs were measured by qPCR. The levels of gene expressions in rat sera were tested 14 days after the last ISO injection. As indicated in Figure 3, higher expression levels of *miR-137* (Figure 3a) and *miR-106b-5p* (Figure 3b) were detected in the ISO/saline rats compared to the control/saline group (*p* < 0.001). Both *miR-137* and *miR-106b-5p* were upregulated by 8.6-fold and 8.7-fold in the ISO/saline group, “HF rats,” respectively, compared to the negative control group, “control/saline” (*p* < 0.0001).

### 2.3. Effect of Cyclocreatine Phosphate on Nourin-Associated miR-137 and miR-106b-5p

As indicated in Figure 3, ISO rats treated with CCrP at a dose of 0.8 g/kg/day for 14 days showed significant reduction of gene expression of *miR-137* by 75% (Figure 3a) (*p* < 0.0001) and of *miR-106b* by 44% (Figure 3b) (*p* < 0.0001). CCrP at a lower dose of 0.4 g/kg/day showed a 33% reduction of *miR-137*, while at the higher dose of 1.2 g/kg/day, it showed a reduction of 68% (Figure 3a). Similarly, CCrP at doses of 0.4 g/kg/day and 1.2 g/kg/day showed a significant reduction of gene expression of *miR-106b-5p* by 18% and 72%, respectively (Figure 3b). As indicated in Figure 3, CCrP reduced expression of Nourin-associated *miR-137* and *miR-106b-5p* in a dose–response manner. Additionally, CCrP treatment of healthy rats at 0.8 g/kg/day for 14 days did not affect gene expression levels of Nourin-associated *miR-137* and *miR-106b-5p* (Figure 3a,b) compared to the saline-treated healthy rats with baseline values, suggesting lack of toxicity in rat hearts. These results are supported by absence of CCrP effect on kidney and liver function (Figure 2b).

In summary, the results support the hypothesis that the expression levels of Nourin-associated *miR-137* and *miR-106b-5p* are markedly increased in ISO/saline “HF rats” which we demonstrated to develop HF [9], while CCrP treatment in ISO/CCrP “non-HF rats” exhibited cardioprotective activity by preventing ischemic injury, normalized the gene level, and prevented the development of HF [9]. These HF results further support our previous findings that CCrP protects against ischemic injury and inhibits the formation and release of the circulating Nourin protein in intact canine models of AMI and cardiopulmonary bypass surgery [6].

## 3. Discussion

The major hallmark of ischemic injury is the pathophysiological cascade including inflammatory response, oxidative stress, apoptosis, and necrosis, which contribute to myocardial injury [1]. MicroRNAs are short single-strand non-coding RNAs sequences which control various physiological and pathological cellular processes through post-transcriptional regulation of gene expression; thereby, the association with different human diseases has been demonstrated [16]. Recent studies have reported a complex relationship between circulating miRNAs and the various aspects of HF such as oxidative stress [17], inflammation, apoptosis, hypertrophy, and fibrosis [18]. Circulating miRNAs have also attracted attention as promising diagnostic and monitoring markers of therapy response in HF patients given their structural stability and non-invasive sampling [19]. It was reported that *miR-145*, *miR-7*, *miR-181*, and *miR-147* inhibited cardiomyocyte apoptosis and that *miR-214* and *miR-29a* are related to fibrosis [19,20], while *miR-155* promotes tissue inflammation [19]. In ischemic HF, it has been demonstrated that the upregulation of *miR-155* induces cardiac inflammation and that *miR-182* promotes apoptosis, while upregulation of *miR-181* inhibits oxidative stress and *miR-214* attenuates cardiomyocyte fibrosis [21]. On the other hand, *miR-147*, *miR-7*, *miR-29b*, and *miR-21* appear to be downregulated [20], while *miR-21* was demonstrated to alleviate apoptosis [22].

Recently, blood-based miRNAs serve as non-invasive diagnostic and therapeutic biomarkers in heart failure [23]. In previous studies, the diagnostic, prognostic, and therapeutic value of *miR-137* has been highlighted [21]. Lok et al. have demonstrated that plasma *miR-137* expression is increased in HF patients and it directly regulates the *alpha 1-antichymotrypsin (ACT)* gene at the post-transcriptional level and that both biomarkers are localized at cardiomyocytes and stromal cells suggesting a role of *miR-137* in reverse myocardial remodeling [24]. Furthermore, it was found that high expression levels of *miR-137* was detected in spontaneously hypertensive rat hearts, in which *miR-137* may promote cardiac remodeling by upregulating *Ang II* and the *TGF-B1*/Smad3 signaling pathway, and that captopril intervention can inhibit *miR-137* expression [25]. Moreover, it was suggested that aberrant expression of *miR-137* not only reflects high blood pressure, but also predicts its severity [23].

In another study, *miR-106b* was identified as one of the miRNA clusters that are associated with age-associated changes. It was reported that altered expression of *miR-106b* significantly affected cardiac function and structure during aging stress [26]. Experimentally, circulating levels of *miR-423-5p* and *miR-106b* were markedly increased in hypertension-induced HF, which was confirmed via RT-qPCR analysis of plasma RNA from hypertensive rats. Additionally, *miR-106b* is upregulated in cardiac tissue of patients with dilated cardiomyopathy and that both *miR-106b* and *miR-15b* modulate apoptosis and angiogenesis in myocardial infarction [19,27]. Recently, a large-scale study was conducted on 2681 HF patients and found that the two-fold increase in plasma miR-106b was associated with incidence of HF [28]. The gene-associated ontology revealed that miR-106b is specified in pathogenesis of HF through direct regulation of transforming growth factor β signaling, cell growth, and apoptosis [27]. On the other hand, *miR-106b* is one of the 55 miRNAs noted to be upregulated in ischemic and non-ischemic cardiomyopathy. In addition, it was demonstrated that plasma *miR-106b*, *miR-93*, and *miR-16* are markedly increased in hypertension-induced HF [19].

Isoproterenol is a beta-adrenergic agonist which elicits myocardial stress in high doses and induces pathological changes in rats through excessive production of oxygen radicals (ROS) which further decreases cardiac contractility and results in myocardial necrosis [17,21,22]. Thus, ISO is one of the most popular experimental non-invasive models to evaluate the cardioprotective effect of natural and pharmacological compounds [29,30]. Levels of serum *miR-21* were positively associated with myocardial infarction size. In post-AMI patients, *miR-21* was significantly correlated with the absolute change in infarction volume, showed a trend for positive correlation with left ventricular (LV) ejection fraction, and was associated with AMI mortality [31]. AMI patients also had significantly higher levels of plasma *miR-21* as compared to healthy controls. Additionally, *miR-21* was shown to be a novel biomarker that was predictive of LV remodeling after AMI, which correlated with several traditional markers of AMI, including CK-MB, creatine kinase (CK), and cardiac troponin I, with comparable diagnostic accuracy, and also as a potential diagnostic biomarker of stable and unstable angina patients [32,33].

Since we have recently demonstrated that CCrP treatment prevents the development of heart failure in the ISO/saline “HF rats” [9], we investigated the expression levels of Nourin-associated miRNAs in these rats and determined the cardioprotective effect of CCrP treatment on myocardial injury and molecular levels in both the ISO/saline “HF rats” and the ISO/CCrP “non-HF rats.” While high CK-MB levels were observed in the ISO/saline “HF rats” 24 h after the last ISO injection indicative of myocardial injury, significant cardioprotection was seen in the ISO/CCrP “non-HF rats” with normal CK-MB levels. After 14 days, gene expression profile showed significant upregulation of *miR-137* and *miR-106b-5p* in the ISO/saline “HF rats” compared to the control/saline group and that CCrP treatment significantly downregulated the expression of Nourin-associated *miR-137* and *miR-106b-5p* in a dose–response manner in the ISO/CCrP “non-HF rats.” Larger studies are needed to further determine the diagnostic and therapeutic roles of Nourin-associated *miR-137* and *miR-106b-5p* in HF. Furthermore, although the current preliminary safety studies indicated that healthy rats treated with CCrP for 14 days showed no toxicity on heart, liver, and renal function, more comprehensive toxicity studies are needed.

## 4. Materials and Methods

### 4.1. Bioinformatics Analysis

Bioinformatics analysis was conducted to analyze differentially expressed genes (DEGs) that are incorporated with ischemic myocardial injury and related to the Nourin amino acid sequence as we previously reported [14].

### 4.2. Experimental ISO Rat Model (ISO/Saline “HF Rats” and ISO/CCrP “Non-HF Rats”)

To determine the Nourin-associated miRNA gene expression levels in experimental heart failure and the cardioprotective effect of CCrP administration, we used the standard ISO rat model [34]. The rats were injected subcutaneously (SC) with isoproterenol hydrochloride (Sigma-Aldrich; Merck KGaA, Darmstadt, Germany) for two consecutive days at doses of 85 and 170 mg/kg/day, respectively, then left for an additional two weeks. Then, the ISO/saline rats were treated with 1 mL saline by intraperitoneal (IP) injection daily for an additional two weeks. The ISO/CCrP rats were treated IP with 1 mL CCrP (Nour Heart, Inc., Vienna, VA, USA) 24 h and 1 h before the first ISO administration, then daily for an additional two weeks. According to our previous studies, CCrP at a dose of 0.8 gm/kg/day is the most effective dose to prevent myocardial ischemic injury and cardiac remodeling, which resulted in restoration of normal ejection fraction, contractile function, and physical activity in intact canine and rat models of AMI, global cardiac arrest, cardiopulmonary bypass, heart transplantation, and HF [6,9,12]. For the current study, a total of 25 male Wistar rats (6–8 weeks old) weighing 180–220 g were purchased from Cairo University Research Park’s Animal Technology Laboratory (Egypt) and caged at controlled temperature (20–25 °C) and humidity (45–55%) with a twelve hours light/dark cycle and free access to food and water. All experimental procedures were approved by the Ethics Committee at the Faculty of Pharmacy, Cairo University (Permit number PT 2733) and were conducted in compliance with the Guide for Care and Use of Laboratory Animals published by the US National Institutes of Health (NIH publication No. 85-23, revised in 2011). The start date of the project was 19 May 2019.

Wistar rats (*n* = 25) were randomly divided into four groups: group I (ISO/saline) (*n* = 6), where ISO rats were treated with IP injections of saline; group II (ISO/CCrP) (*n* = 5), where ISO rats were treated with IP injections of CCrP at 0.8 g/kg/day; group III (control/saline) (*n* = 5), where healthy rats were treated with IP injections of saline; and group IV (control/CCrP) (*n* = 4), where healthy rats were treated with IP injections of CCrP at 0.8 g/kg/day for 14 days to evaluate potential drug toxicity. Because of limited drug availability, the cardioprotective activity of CCrP was tested using CCrP at a lower dose of 0.4 g/kg/day (*n* = 3) and a higher dose of 1.2 g/kg/day (*n* = 2).

### 4.3. Biochemical Assessment

Twenty-four hours after the last ISO injection, serum samples were collected to measure CK-MB levels using a Rat Creatine Kinase MB isoenzyme ELISA Kit (cat. No. DEIA-FN285; Creative Diagnostics, New York, NY, USA). The test was performed according to the manufacturer’s instructions. In addition, fourteen days after the last ISO injection, the renal function was evaluated by measuring the serum level of urea and creatinine using the endpoint colorimetric assays, as well as the alanine transaminase (ALT) serum level in healthy rats injected IP daily with CCrP 0.8 g/kg/day for 14 days. At the end of the study (fourteen days after the last ISO injection), the rats were anesthetized with 30 mg/kg pentobarbital sodium and sacrificed by decollation. Three to five mL of peripheral blood were collected in sterile vacutainer tubes, centrifuged at 5000× *g* for 10 min at 4 °C to avoid degradation of RNAs and proteins; then the serum was removed and immediately stored at −80 °C until analysis.

### 4.4. Molecular Assessment of Nourin-Associated miR-137 and miR-106b-5p

Total RNAs and miRNAs were extracted from sera samples using an RNeasy Mini Kit (Qiagen, Hilden, Germany) according to the manufacturer’s protocol, and RNA concentration and purity were evaluated spectrophotometrically at 260 and 280 nm. RNA integrity was visually confirmed by agarose gel electrophoresis. Synthesis of cDNA was completed by reverse transcription reaction using a miScript RT Kit (Qiagen, Hilden, Germany). In the second step, cDNA was amplified for miRNA expression using a miScript Primer Assay for miRNA amplification (Rn_*miR-137*_1 and Rn_*miR-106b-5p*_1 miScript Primer Assay). The At_U19_1 and Rn_ GAPDH genes were used as reference housekeeper genes. The thermal cycling was adjusted according to the manufacturer’s instructions. The PCR analysis was conducted on a Rotor-Gene Q 5plex high resolution melting (HRM) platform (Qiagen, Hilden, Germany). The fluorescence data were collected at the extension step. Following amplification, gene expression was calculated using the 2^∆∆Ct^ method.

### 4.5. Statistical Analysis

Quantitative values were expressed as the means ± SDs and range. The significant difference for the measured variables between different experimental groups was analyzed using GraphPad Prism (version: 8; GraphPad Software Inc., San Diego, CA, USA). The analysis of variances test (ANOVA) followed by the Tukey’s post-hoc test was used to compare multigroup results. The *p*-value < 0.05 was considered statistically significant.

## 5. Conclusions

Although the immune system plays a significant role in cardiac healing after ischemic injury, its persistent activation is reported to participate in the pathogenesis of heart failure, cardiac fibrosis, left ventricular remodeling, as well as in the reduction of ejection fraction and contractile dysfunction. The results of this study indicate that the Nourin-associated *miR-137* and *miR-106b-5p* are novel inflammatory markers in the pathogenesis of heart failure in the ISO/saline “HF rats” and that the administration of the bioenergetic CCrP prevents ischemic injury and maintains the baseline gene expression levels of Nourin miRNAs after 14 days in the ISO/CCrP “non-HF rats.” Inhibition of Nourin-associated miRNAs by CCrP might likely play a protective role by controlling early reperfusion inflammation-induced injury, resulting in the reduction of cardiac inflammation and remodeling, as well as in the restoration of normal ejection fraction [9]. We, therefore, believe that CCrP is a promising first-in-class cardioprotective therapy that can be used as an antagonist to Nourin to control early ischemia-induced cardiac inflammatory response without affecting the healing process. Additionally, the gene-based cardiac Nourin blood test may potentially be used as a novel monitoring marker of drug therapy response in heart failure patients where a continued high gene expression level of Nourin-associated *miR-137* and *miR-106b-5p* after therapy would likely be indicative of an unsuccessful therapy and/or drug-induced cardiotoxicity/myocardial injury, while a drop in the Nourin gene expression level would likely be indicative of a successful therapy and an improvement in heart health. Further studies are needed to determine the clinical utility of Nourin-associated miRNAs as monitoring markers in heart failure and the therapeutic potential of CCrP in addition to its preventive therapy.

## Data Availability

Not applicable.

## Abbreviations

AMI	acute myocardial infarction
ACT	alpha 1-antichymotrypsin
ALT	alanine transaminases
CK-MB	creatine kinase-myocardial band
CCr	cyclocreatine
CCrP	cyclocreatine phosphate
EF	ejection fraction
DEGs	differentially expressed genes
ELAM-1	endothelial cell leukocyte adhesion molecule-1
GO	gene ontology
HF	heart failure
HRM	High resolution melting
ICAM-1	intercellular adhesion molecule 1
IL-6	interleukin 6
IL-8	interleukin 8
IL-1β	interleukin 1β
ISO	isoproterenol
KEGG	Kyoto Encyclopedia of Genes and Genomes
LECAM-1	leukocyte-endothelial cell adhesion molecule 1
LV	left ventricular
miR	microRNA
MI	myocardial injury
STEMI	ST elevation myocardial infarction
TNF-α	tumor necrosis factor alpha
UA	unstable angina
VECs	vascular endothelial cells
LFA-1	lymphocyte function-associated antigen
